# Lack of Durable Cross-Neutralizing Antibodies Against Zika Virus from Dengue Virus Infection

**DOI:** 10.3201/eid2305.161630

**Published:** 2017-05

**Authors:** Matthew H. Collins, Eileen McGowan, Ramesh Jadi, Ellen Young, Cesar A. Lopez, Ralph S. Baric, Helen M. Lazear, Aravinda M. de Silva

**Affiliations:** University of North Carolina, Chapel Hill, North Carolina, USA

**Keywords:** viruses, Zika virus, dengue virus, cross-neutralizing antibodies, vector-borne infections, flaviviruses, DENV

## Abstract

Cross-reactive antibodies elicited by dengue virus (DENV) infection might affect Zika virus infection and confound serologic tests. Recent data demonstrate neutralization of Zika virus by monoclonal antibodies or human serum collected early after DENV infection. Whether this finding is true in late DENV convalescence (>6 months after infection) is unknown. We studied late convalescent serum samples from persons with prior DENV or Zika virus exposure. Despite extensive cross-reactivity in IgG binding, Zika virus neutralization was not observed among primary DENV infections. We observed low-frequency (23%) Zika virus cross-neutralization in repeat DENV infections. DENV-immune persons who had Zika virus as a secondary infection had distinct populations of antibodies that neutralized DENVs and Zika virus, as shown by DENV-reactive antibody depletion experiments. These data suggest that most DENV infections do not induce durable, high-level Zika virus cross-neutralizing antibodies. Zika virus–specific antibody populations develop after Zika virus infection irrespective of prior DENV immunity.

Zika virus is a member of the flavivirus family of arthropodborne viruses, which includes West Nile virus, yellow fever virus, tick-borne encephalitis virus, and dengue virus (DENV) (*1*). The recent emergence of Zika virus in the Western Hemisphere caused widespread international concern. Although Zika virus infection is most commonly asymptomatic or causes only a mild, self-limited illness, recent epidemics have revealed new manifestations of Zika virus disease, including Guillain-Barré syndrome (*2*,*3*) and sexual transmission (*4*). Most alarmingly, and distinct from other flaviviruses, Zika virus infection during pregnancy can result in a spectrum of developmental abnormalities (congenital Zika syndrome) (*5*), which can include ocular damage, microcephaly, and fetal death (*6*). These manifestations raise public health challenges unique from those of other vectorborne diseases, particularly preventing sexual transmission and protecting pregnant women.

Given shared ecology and mosquito vectors, Zika virus is emerging in areas with endemic DENV transmission. In many areas of Latin America, most persons has been exposed to >1 natural DENV infections (*7*); in some regions, DENV vaccination has been implemented or is in clinical trials (*8*). Because there is known serologic cross-reactivity between Zika virus and DENV (*9*), determining how DENV immunity affects subsequent Zika virus infection is important.

The ability of flavivirus infection to induce antibodies that are virus-specific and that cross-react with different flaviviruses is well known (*10*–*13*), and flaviviruses have been grouped as serocomplexes on the basis of degree of antigenic overlap (*11*). Zika virus has not been studied extensively in this context; although it shares 54%–58% of its nucleotide sequence with the 4 serotypes of DENV in the envelope protein coding sequence (*14*), the extent to which Zika virus will group antigenically with the DENV serocomplex is unclear. Several groups recently reported that antibodies isolated from persons with prior DENV infections cross-neutralize Zika virus and cross-protect in animal models of Zika virus infection (*15*–*19*). These results raise the possibility of DENV infections or vaccines cross-protecting against Zika virus. Moreover, researchers have been concerned that the plaque/focus reduction neutralization test, the standard serologic assay for distinguishing different flavivirus serocomplexes, will be unable to differentiate Zika virus from DENV. During 2016 in North Carolina, USA, we studied whether persons exposed to DENV maintain cross-neutralizing antibodies to Zika virus.

## Methods

### Human Subjects and Immune Serum

Serum was collected from North Carolina residents who had probable or confirmed DENV or Zika virus infection on the basis of self-reported symptoms and travel to areas in which these viruses are endemic. Serum samples from this study were assigned consecutive and arbitrary identification numbers such as DT165. A subset of DENV immune serum was obtained from a reference panel distributed by the Pediatric Dengue Vaccine Initiative. Samples were tested by virus-capture ELISA, and DENV- or Zika virus–reactive serum was further characterized by neutralization assays on Vero cells. All donations were collected in compliance with the Institutional Review Board of the University of North Carolina at Chapel Hill (protocol 08–0895).

Serum that had neutralizing antibodies to 1 DENV serotype or to Zika virus with minimal cross-neutralizing antibodies were defined as primary flavivirus infections (meaning that the 50% inhibitory concentration [IC_50_] for a single DENV serotype or Zika virus was >4-fold higher than for any other virus tested). In most cases, the person’s travel history corroborated the primary immune status. Serum that had high levels of neutralizing antibody to >2 flaviviruses were defined as secondary (repeat) flavivirus infections. Most secondary infection samples were from persons who had resided in DENV- or Zika virus–endemic countries for >5 years.

### Viruses and Cells

#### Zika Virus Stocks

The MR766 and Dakar 41519 strains of Zika virus were obtained from R. Tesh (World Reference Center for Emerging Viruses and Arboviruses, University of Texas Medical Branch, Galveston, TX, USA) (*20*,*21*). The Centers for Disease Control and Prevention (Atlanta, GA, USA) provided Zika virus strains H/PF/2013 and PRVABC59 (*22*,*23*).

#### DENV Stocks

All in vitro assays were conducted with the DENV World Health Organization reference strains: DENV-1 West Pac 74, DENV-2 S-16803, DENV-3 CH54389, and DENV-4 TVP-360 (initially obtained from R. Putnak, Walter Reed Army Institute of Research, Silver Spring, MD, USA). Virus stocks were prepared in C6/36 *Aedes albopictus* mosquito cells (ATCC no. CRL-1660) or Vero *Cercopithecus aethiops* monkey cells (ATCC no. CCL-81). C6/36 cells were grown at 32°C with 5% CO_2_ in minimum essential medium supplemented with 10% fetal bovine serum (FBS), L-glutamine, nonessential amino acids, and HEPES (2-hydroxyethyl)-1-piperazineethanesulfonic acid) buffer. Vero cells were grown at 37°C with 5% CO_2_ in Dulbecco modified Eagle medium supplemented with 5% FBS and L-glutamine. Virus stocks were titrated on Vero cells by plaque assay or focus-forming assay (*24*). Infected cell foci were detected at 40–48 h after infection, after fixation with 1%–2% paraformaldehyde and incubation with 500 ng/mL of flavivirus cross-reactive mouse monoclonal antibody (mAb) E60 (*25*), 2H2 (*26*), and/or 4G2 (*26*). After incubation with a 1:2,000 dilution of horseradish peroxidase–conjugated goat antimouse IgG (Sigma, St. Louis, MO, USA), foci were detected by addition of TrueBlue substrate (KPL). We analyzed foci with a CTL Immunospot instrument (CTL, Cleveland, OH, USA). All studies were conducted under biosafety level 2 containment.

### ELISA

We measured binding of human serum IgG to DENV or Zika virus by ELISA as previously described (*27*). In brief, DENV virions were captured by the anti-E protein mouse mAb 4G2, blocked with 3% normal goat serum (GIBCO Life Technologies, Carlsbad, CA, USA) or 3% nonfat dairy milk (LabScientific, Inc., Highlands, NJ, USA), and incubated with human serum at indicated dilutions at 37°C for 1 h, and binding was detected with an alkaline phosphatase–conjugated antihuman secondary antibody (Sigma) and *p*-nitrophenyl phosphate substrate (Sigma). Absorbance at 405 nm was measured on an Epoch plate reader (BioTek, Suwanee, GA, USA). ELISAs used to confirm depletion were performed as given earlier, with the exception that 50 ng purified DENV was coated directly to the plate at 37°C for 1 h at 1:50 dilution before serum was tested. ELISA data are reported as optical density values that are the average of technical replicates, unless otherwise indicated. The average optical density for technical replicates using naive human serum at the same dilution factor as test samples serves as the negative control in ELISAs. In depletion experiments, the OD of depleted sample is expressed as percentage of control from same serum for some graphs as indicated.

### Neutralization Assays

We adapted the previously described focus-reduction neutralization test (FRNT) (*28*) to a 96-well format (*18*). Neutralization titers were determined by FRNT by serial 3-fold dilution of human serum and mixing with ≈50–100 focus-forming units of virus in Dulbecco modified Eagle medium with 2% FBS. The virus–antibody mixtures were incubated for 1 h at 37°C and then transferred to a monolayer of Vero cells for titration by focus assay as described earlier. For neutralization assays, we calculated IC_50_ values by using the sigmoidal dose response (variable slope) equation of Prism 6 (GraphPad Software, San Diego, CA, USA). One set of DENV-1–4 neutralization values (DT003) was determined previously by U937 flow-based assay (*29*), but Zika virus neutralization was determined by Vero FRNT. IC_50_ values shown for certain Zika virus-immune serum samples were determined on 24-well plaque assay (*30*) (Table 1). Reported values were required to have an *R^2^* >0.75, a hill slope >0.5, and an IC_50_ within the range of the assay.

**Table 1:** DENV and Zika virus neutralization profiles for persons with travel history to Zika virus–endemic areas*

Serostatus, serum sample ID	Place of Infection	IC_50_†				
		DENV-1	DENV-2	DENV-3	DENV-4	Zika virus
Primary DENV-1						
147	Latin America	3,552	287	557	75	<20
153	Latin America	757	<20	<20	<20	<20
05/262	Asia	274	<20	<20	<20	<20
06/125	Asia	3,823	222	125	80	<20
99/1230	Asia	1,219	63	30	24	<20
Primary DENV-2						
001	Asia	49	2,188	48	89	<20
08/90	Asia	<20	2,966	<20	<20	<20
08/91	Asia	<20	838	<20	<20	<20
09/165	Asia	<20	2,093	<20	<20	<20
09/251	Asia	<20	417	<20	<20	<20
Primary DENV-3						
116	Asia	200	979	5,342	290	<20
118	Latin America	173	374	3,041	56	<20
125	Latin America	99	97	1,648	35	<20
133	Latin America	89	171	3,348	83	<20
06/297	Asia	27	<20	573	<20	<20
Primary DENV-4						
112	Latin America	908	1367	591	18,408	<20
06/105	Asia	<20	<20	<20	941	<20
06/302	Asia	<20	<20	<20	4,130	<20
09/159	Asia	115	226	478	5,694	<20
Primary Zika virus						
168	Latin America	36^ǂ^	<20	78‡	<20	1,382
172	Latin America	<20	<20	<20	<20	8,468
Secondary DENV						
000	Asia	3,306	2,087	1,162	782	<20
003	Asia	556	178	299	<20	146
115	Asia	100	355	830	245	<20
141	Latin America	1,902	1,953	4,530	664	<20
144	Asia	155	191	5,782	1,612	699
145	Asia	601	1,262	240	60	<20
146	Asia	403	1,052	1,480	451	28
155	Asia	215	299	71	27	<20
160	Latin America	947	3,564	131	1,600	<20
06/123	Asia	1,776	827	82	157	<20
06/124	Asia	1,454	1,208	1,673	1,011	<20
09/157	Asia	282	1,104	73	134	<20
09/250	Asia	375	1475	151	94	<20
Secondary Zika virus						
165	Latin America	60‡	79‡	70‡	508	1655
166	Latin America	929	393	4344	240	1814

*DENV, dengue virus; IC_50_, 50% inhibitory concentration; ID, identification. †All neutralization testing performed on Vero cells with the exception of DT003, which was limiting and historic values performed on a U937 Flow-based assay are shown for DENV-1–4. Zika virus IC_50_ values for DT003 are from Vero assays. ‡Curves that did not pass quality metrics are marked. Such curves might signify very low titer IC_50_ values or variable background at the lower limit of detection in serum with no specific neutralization activity.

### Depletions

As previously described (*31*), purified viral antigen for depletions was obtained by infecting Vero cell cultures in 850 cm^2^ roller bottles (Greiner Bio-One, Monroe, NC, USA) with DENV and then concentrating DENV-containing supernatants at 4°C by tangential flow ultrafiltration using the Pellicon mini system with a 100-kD cutoff membrane (Pellicon-2 mini Holder and Pellicon-2 Mini Filters; Millipore, Darmstadt, Germany). The flow rate was 400 mL/min, and filtration rate was ≈100 mL/min; pressure was 20–30 psi. Concentrated virus was then purified on a 15%–65% sucrose gradient by ultracentrifugation (SW 40 Ti, Beckman Coulter, Brea, CA, USA) at 21,583 relative centrifugal force for 18 h at 4°C. The fractions with maximal content of virus was determined by resolving fractions by SDS-PAGE and protein concentration was measured by Micro BCA Protein Assay Kit (Thermo Fisher Scientific, Waltham, MA, USA).

Purified viral antigen was conjugated to Polybeads polystyrene 4.5-μ microspheres (PolyScience, Niles, IL, USA) in accordance with the manufacturer’s instructions (100 μg/250 μL beads) by incubating overnight at room temperature. Control beads were incubated with equal amount of bovine serum albumin. Beads were blocked with 10 mg/mL bovine serum albumin, washed 3 times with 0.1 M borate buffer (pH 8.5), followed by 3 times with phosphate buffered saline. For depletion, serum was diluted 1:10 in phosphate buffered saline and incubated with 100 μg DENV-1 + 100 μg DENV-2 divided over 3 rounds at 37°C for 1 h each. After incubation, tubes were centrifuged at 20,800 relative centrifugal force to pellet beads with bound antibodies, and serum was pipetted off the undisturbed pellet and transferred to new vials. We confirmed depletion efficacy with direct binding ELISA. Serum with higher titers of binding antibodies was subjected to additional rounds of depletion until IgG binding was reduced to background levels.

## Results

To study human antibody interactions between DENV and Zika virus, we assembled 36 late convalescent serum samples from persons exposed to DENV, Zika virus, or both (Table 1). The panel comprised serum from 21 persons exposed to primary flavivirus infections (with each DENV serotype represented and 2 cases of Zika virus) and serum from 15 persons exposed to >2 flavivirus infections, including 2 persons exposed to both DENV and Zika virus.

**Figure 1:**
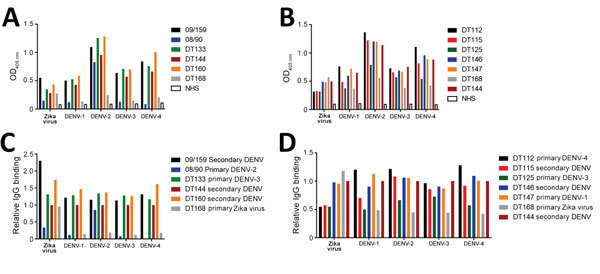
Binding of DENV immune serum to Zika virus virions. Zika virus and 4 DENV serotypes were captured by using plate-bound mouse monoclonal antibody 4G2 and incubated with serum from donors who had had a primary DENV, secondary DENV, or primary Zika virus infection. In 2 separate experiments (A, B), serum binding was detected by using a horseradish peroxidase–conjugated human IgG. C, D) Differential global binding of each virus was accounted for by subtracting background from native human serum and normalizing to a high binding serum common to both plates (DT144). DENV, dengue virus; NHS, naive human serum; OD, optical density.

We measured total IgG binding to DENV and Zika virus using a virus capture ELISA. We observed extensive cross-reactivity between DENV serotypes and between DENV and Zika virus, confirming that cross-reactive binding antibodies are maintained for many years after infection (Figure 1). Although cross-reactive binding antibodies are commonly detected in flavivirus-immune serum, neutralization assays are more specific and can distinguish between previous exposure to various flaviviruses or even between different DENV serotypes (*32*). We therefore tested whether convalescent serum antibodies in persons exposed to DENV cross-neutralize Zika virus by a Vero cell–based neutralization assay. Serum from persons exposed to primary DENV infection of any serotype did not cross-neutralize Zika virus (Table 1; Technical Appendix Figure 1). In contrast, Zika virus was readily neutralized by serum from persons who had traveled to Brazil (DT168) and Colombia (DT172) in 2015 and experienced acute illnesses consistent with an arbovirus infection. Both serum samples strongly neutralized Zika virus, showing low or no cross-neutralization of the 4 DENV serotypes (Table 1; Technical Appendix Figure 1, panels C, D), consistent with primary Zika virus infection.

Persons exposed to secondary DENV infections develop broadly neutralizing antibodies that neutralize even DENV serotypes not encountered by that person (*33*). We determined the neutralization profile for 13 serum samples from persons with secondary DENV. Although the potency of neutralization varied, all but 1 (DT155) of the 13 samples had high levels of neutralizing activity (IC_50_ >100) against at least 3 DENV serotypes, and 7 were highly neutralizing against all 4 serotypes (Table 1). Despite this extensive cross-reactivity, most (10 [77%]) secondary DENV-immune serum samples failed to neutralize Zika virus (Table 1; Technical Appendix Figure 1). Serum from 1 person showed low levels of cross-neutralizing activity (DT146), and 2 serum samples (DT003 and DT144) had high levels of Zika virus neutralizing antibodies. Two donors (DT165 and 166) reported suspected Zika virus infections acquired while in Brazil in 2015 during a known Zika virus outbreak. Serum from these 2 persons also neutralized Zika virus and >1 DENV serotypes, indicating that these most likely represent secondary Zika virus infections (Technical Appendix Figure 1, panels E, F). These results demonstrate that the broadly cross-neutralizing antibody response that is a hallmark of repeat DENV infections is mainly confined to the DENV serocomplex, although in some cases these antibodies also might cross-neutralize Zika virus.

Zika virus strains are divided into 2 genotypes, Asian and African; contemporary Zika virus isolates from Latin America are uniformly of the Asian genotype, consistent with the model that Zika virus spread from Southeast Asia to Oceania and from there to Brazil (*34*). To evaluate the effect of Zika virus strain variation on cross-neutralization, we tested selected DENV and Zika virus–immune serum against 4 Zika virus strains representing diverse temporal and geographic origins (Table 2). All 4 strains exhibited similar neutralization patterns, specifically that they were neutralized by serum from primary (DT168 and DT172) or secondary (DT165 and DT166) Zika virus cases, but not by serum from a secondary DENV infection (DT145) (Table 2; Technical Appendix Figures 2, 3), supporting the idea that Zika virus exists as a single serotype. Zika virus strain MR766 was relatively more susceptible than other strains to neutralization, and it was the only strain to exhibit even low-level neutralization by secondary DENV serum (IC_50_ 67). In general, IC_50_ titers were similar for primary Zika virus serum and for secondary Zika virus serum, consistent with the idea that cross-reactive antibodies from prior DENV infection do not contribute to Zika virus neutralization.

**Table 2:** Zika virus stains and neutralization in selected serum*

Strain	Genotype	Origin	Year	Reference	Serum sample ID, IC_50_			
					DT168	DT172	DT165	DT166
MR766	African	Uganda	1947	(*21*)	2,546	2,898	1,918	3,890
DAK41519	African	Senegal	1982	(*22*)	700	1,186	547	1,203
H/PF/2013	Asian	Tahiti	2013	(*23*)	609	531	516	469
PRVABC59	Asian	Puerto Rico	2015	(*24*)	436	1,606	Not tested	Not tested

*IC_50_, 50% inhibitory concentration; ID, identification.

**Figure 2:**
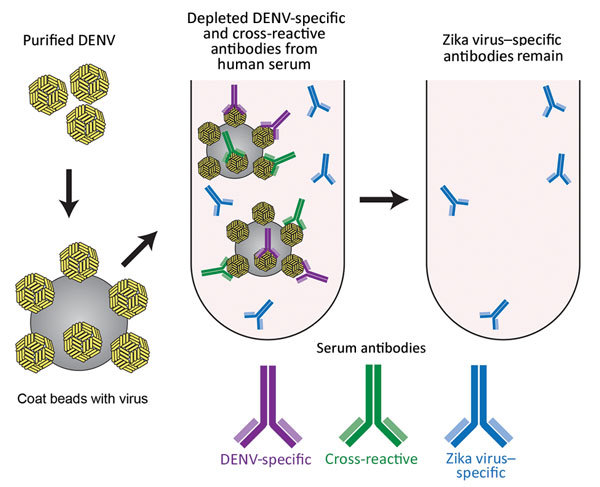
Schematic of the method used for depletion of human serum with DENV antigen to distinguish Zika virus–specific from cross-reactive flavivirus antibodies. Serum was incubated with DENV-1 and DENV-2 coated on polystyrene beads, enabling removal of DENV-specific and cross-reactive antibodies. DENV, dengue virus.

**Figure 3:**
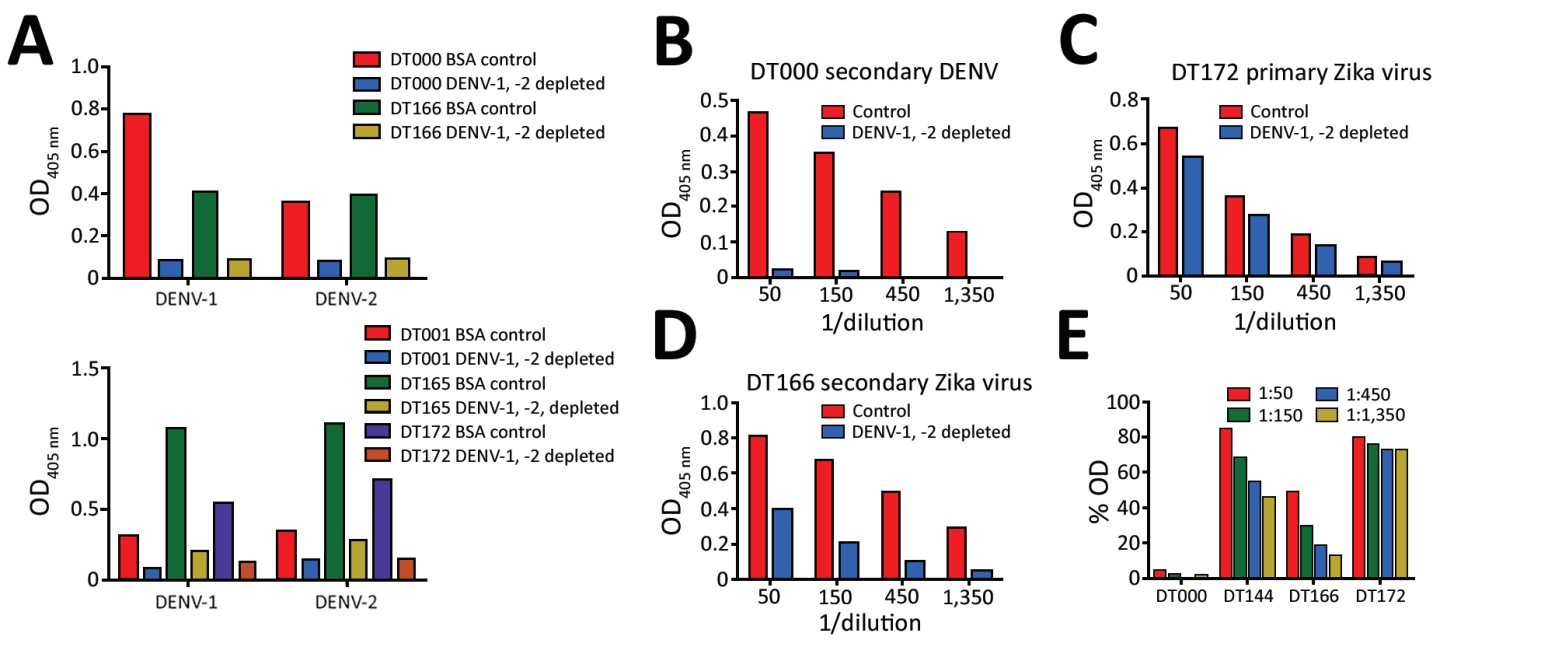
Use of depletion of human serum with DENV antigen to distinguish Zika virus–specific from cross-reactive flavivirus antibodies. A) Depletion efficiency was determined by direct ELISA. Plates were coated with depleting antigens (DENV-1 and DENV-2) and binding of control and depleted serum was measured. B–E) Binding of depleted serum to Zika virus H/PF/2013 was measured by capture ELISA. BSA, bovine serum albumin; DENV, dengue virus; OD, optical density.

**Figure 4:**
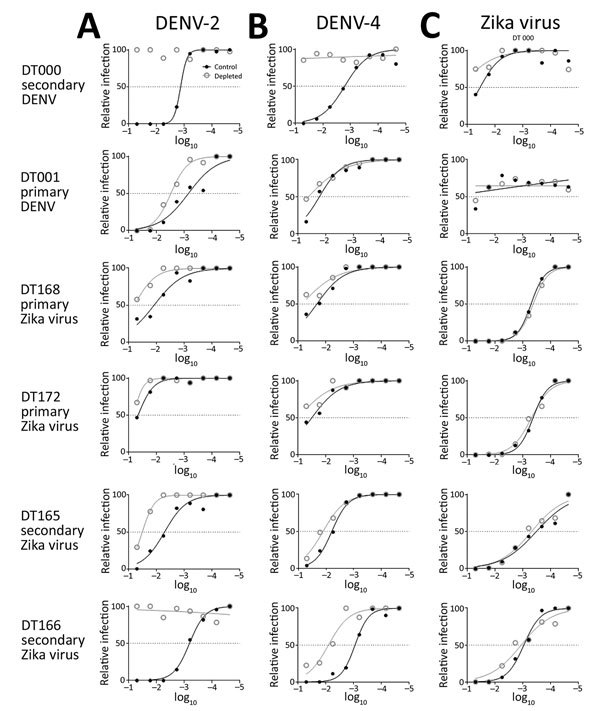
Lack of contribution of cross-reactive DENV antibodies to Zika virus neutralization. Immune serum was depleted with DENV-1 and DENV-2 antigens bound to polystyrene beads, and neutralization activity was measured against DENV-2 (A), DENV-4 (B), and Zika virus (C) for indicated serum. DENV, dengue virus. Dilutions are 1:the value given.

To test the hypothesis that Zika virus infection elicits type-specific antibody, even in the presence of DENV immunity, we incubated serum with polystyrene beads coated with purified DENV antigen to remove DENV-specific and flavivirus cross-reactive antibody (Figure 2, Table 3). We then assessed binding and neutralization of Zika virus by the depleted serum. IgG binding to DENV-1 and DENV-2 antigen was lower in depleted serum than in control serum, confirming success of this method (Figure 3, panel A). IgG binding to captured Zika virus was lower when DENV-immune serum was depleted, but depletion only partially reduced binding from secondary Zika virus serum and had little effect on serum from primary Zika virus infections (Figure 3, panels B–E). Depletion successfully removed DENV neutralizing antibodies because depleted serum from primary DENV-2 (DT001), secondary DENV (DT000), and secondary Zika virus (DT165 and 166) cases all exhibited marked reductions in ability to neutralize DENV-2 (Figure 4, Table 3). Also, broadly neutralizing serum lost the ability to neutralize a heterologous DENV serotype (DENV-4) after depletion, establishing that cross-neutralizing antibodies were effectively removed from these sera (Figure 4, Table 3). Zika virus neutralization activity was entirely maintained after DENV depletion of serum from persons with primary Zika virus (DT168 and 172) and mostly preserved in depleted serum from persons with secondary Zika virus (DT165 and 166), even when neutralization activity was lost to all DENV serotypes tested (Figure 4, Table 3). 

**Table 3:** 50% Inhibitory concentrations for serum in depletion experiments of DENV and Zika virus*

Serum sample ID	DENV-2	DENV-4	Zika virus
DT000			
Control	744	612	29
Depleted	<20	<20	<20
DT001			
Control	1,376	60	<20
Depleted	331	24	<20
DT168			
Control	83	48	1,978
Depleted	<20	<20	2,351
DT172			
Control	22	32	2,341
Depleted	<20	<20	2,046
DT165			
Control	198	162	3,067
Depleted	31	78	2,060
DT166			
Control	1,556	1,138	1,090
Depleted	<20	126	873

*DENV, dengue virus; ID, identification. Specific antibodies were depleted using beads coated with DENV1 and DENV2 antigens. Beads coated with BSA were used as a control.

## Discussion

Because Zika virus is emerging in areas with high rates of dengue prevalence, the extent of antibody cross-reactivity between these 2 viruses needs to be thoroughly evaluated. Recent studies have reported that DENV infection results in antibodies that cross-neutralize Zika virus (*15*,*16*,*18*). Plasmablasts isolated from patients during or immediately after recovery from acute DENV infection produced antibodies that cross-neutralized Zika virus in cell culture (*35*) and were protective in a mouse model of Zika virus infection (*18*). Priyamvada et al. (*16*) demonstrated moderate to high-titer Zika virus neutralization in serum from 9 DENV-infected persons; Zika virus neutralization activity was maintained out to 100 days of convalescence in 1 of the 5 persons with paired samples. They further reported that 7 of 47 mAbs derived from plasmablasts from 4 patients with acute DENV infection cross-neutralized Zika virus.

Our data do not demonstrate frequent and high-level cross-neutralization of Zika virus after exposure to DENV. Of 19 persons who had recovered from primary DENV infections, none showed cross-neutralization of Zika virus. Among persons exposed to repeat DENV infections, 3 (23%) of 13 showed Zika virus neutralizing antibodies. The remaining 10 persons had no detectable Zika virus neutralizing antibodies despite having high levels of neutralizing antibodies to multiple DENV serotypes. DT165 and 166 are classified as secondary Zika virus strains on the basis of neutralization profile, epidemiologic context (these donors had fever and rash illness while residing in northeastern Brazil in 2015 during peak Zika virus transmission), and the presence of Zika virus type–specific antibodies in their serum. Accordingly, these persons have high titers of neutralizing antibodies to Zika virus in addition to neutralization activity to >1 DENV serotypes. These results are consistent with those reported by Swanstrom et al., who found that only 1 of 16 persons exposed to repeat DENV infections had Zika virus IC_50_ values >1:100 (*18*). Low-level cross-neutralization to heterologous DENV serotypes is also observed into late convalescence after primary DENV infection; however, this phenomenon typically does not preclude accurate diagnosis of the originally infecting DENV serotype, nor does it confer immunity to secondary DENV infection by heterologous serotypes (*30*,*36*).

A key feature of acute DENV infection is the transient induction of high levels of flavivirus cross-neutralizing and cross-protective antibody (*33*). Over a few months, the neutralizing antibody response becomes more specific to >1 DENV serotypes, with little to no cross-neutralization of viruses belonging to other flavivirus serocomplexes (*33*). Timing of sample collection probably explains high levels of DENV–Zika virus cross-neutralizing antibodies observed by only some groups. Barba-Spaeth et al. (*15*) and Priyamvada et al. (*16*) performed their studies with blood samples collected from patients a few days to weeks after recovery from acute DENV infection, when overall antibody response is known to be broadly cross-neutralizing. In contrast, our studies were performed with samples collected >6 months after infection when the neutralizing antibody response has become more specific to the viruses responsible for infection.

In essence, the question is whether Zika virus will behave as a “fifth serotype” of DENV or as a flavivirus outside the DENV serocomplex. Extensive cross-reactivity in IgG binding between these viruses clearly occurs, consistent with sequence homology and structural similarity among different flavivirus serocomplexes (*10*,*12*). However, binding does not reliably predict functional outcomes, such as neutralization, enhancement, or protection, and our results suggest that most persons infected with DENVs do not maintain high levels of Zika virus neutralizing antibodies 6 months after DENV infection. Although Zika virus is genetically more closely related to the DENVs than to other flaviviruses, the long-lived antibody cross-neutralization within the DENV serocomplex does not extend to Zika virus in most of our cohort. Furthermore, our data indicate that a population of Zika virus–specific neutralizing antibodies develops after Zika virus infection, even in the presence of preexisting DENV immunity. The corollary is that cross-reactive antibodies and memory B cells from the prior DENV infection are not the major source of Zika virus neutralizing antibodies; rather, de novo priming of naive B cells by Zika elicits Zika type-specific antibody responses. Thus, we propose that Zika virus does not belong to the DENV serocomplex and that DENV-immune persons will remain susceptible to Zika virus infection. Moreover, live attenuated tetravalent DENV vaccines are being evaluated for safety and efficacy in Asia and Latin America. Our results indicate that DENV neutralizing antibody induced by these vaccines are unlikely to protect against Zika virus infection.

In the field, undifferentiated fever in the tropics can have a multitude of causes and often presents a diagnostic challenge (*37*). Molecular detection of Zika virus has good specificity but is not ideal for diagnosing it in persons who seek care many days after illness onset or have asymptomatic infection. Simple serologic assays based on binding antibody are difficult to interpret because of flavivirus cross-reactivity (*9*,*10*). This scenario, typified by DT144, 165, and 166, whereby flavivirus exposure history is uncertain with currently available assays (IgG or IgM ELISA), represents a critical challenge facing public health systems throughout the tropics where billions of persons are at risk for DENV and Zika virus infections (*38*). Our results suggest that the classical flavivirus plaque/focus reduction neutralization test might be reliable for determining previous Zika virus infection, particularly in the setting of retrospective serologic surveys and vaccine trials. We also note that the neutralization test is not reliable for testing samples collected during or soon after recovery from a DENV or Zika virus infection because of temporarily broad cross-neutralization and poor specificity. Indeed, US public health laboratories and others have reported on the poor specificity of testing for Zika virus in acute or early convalescent samples using currently available tests and algorithms (*39**–**42*). On the basis of our studies using samples collected at late convalescence, we propose that the neutralization assay might retain utility for supporting Zika virus and DENV vaccine and other clinical trials; population-level serosurveillance; and clinical management of some patients, such as pregnant women, when blood samples are collected many months after a suspected infection.

Interest and investigation are ongoing regarding whether Zika virus strain–dependent factors might explain phenomena observed during the current epidemic. Although a more comprehensive and in-depth analysis of Zika virus genetic variation might reveal viral determinants of pathogenesis, our experiments suggest that epitopes conferring susceptibility to neutralization by human serum have not changed substantially over time. We do find that the prototype Zika virus strain MR766 is more readily neutralized than other Zika virus strains. MR766 might provide the most sensitive screen for cross-neutralizing antibody, but relevance of such antibody should be confirmed by an isolate more representative of contemporary circulating viruses.

In conclusion, the current Zika virus epidemic presents many urgent challenges but also great opportunities to dramatically expand our knowledge of humoral immunity to flaviviruses. Although our results argue for infrequent cross-neutralization of Zika virus by DENV-immune serum and for development of independent populations of neutralizing antibody to these 2 viruses, additional and larger studies are needed to determine whether the rate of Zika virus cross-neutralization varies in different populations, particularly in DENV-endemic areas where ongoing subclinical exposure to DENV could further broaden the range of cross-neutralization in polyclonal serum. That effective vaccines have been developed against several flaviviruses is encouraging (*43**–**46*), but much remains to be learned about Zika virus–specific antibody responses and the dynamics of cross-reactive antibody in persons with multiple flavivirus exposures. Knowledge on these fronts will better inform Zika virus vaccine development, rational design of serodiagnostic tests, and general understanding of antibody responses to related viruses.

Technical Appendix Limited cross-neutralization between Zika virus; dengue virus and different Zika virus strains with similar neutralization profiles.

## References

[1] Lazear HM, Diamond MS. Zika virus: new clinical syndromes and its emergence in the Western Hemisphere. J Virol. 2016;90:4864–75. 10.1128/JVI.00252-16 26962217 PMC4859708

[2] Smith DW, Mackenzie J. Zika virus and Guillain-Barré syndrome: another viral cause to add to the list. Lancet. 2016;387:1486–8. 10.1016/S0140-6736(16)00564-X 26948432

[3] Oehler E, Watrin L, Larre P, Leparc-Goffart I, Lastere S, Valour F, et al. Zika virus infection complicated by Guillain-Barre syndrome—case report, French Polynesia, December 2013. Euro Surveill. 2014;19:20720. 10.2807/1560-7917.ES2014.19.9.20720 24626205

[4] Moreira J, Peixoto TM, Machado De Siqueira A, Lamas CC. Sexually acquired Zika virus: a systematic review. Clin Microbiol Infect. 2017 Jan 3 [Epub ahead of print]. pii: S1198-743X(16)30659-0. **PMID: 28062314** 10.1016/j.cmi.2016.12.027 28062314

[5] Miranda-Filho DB, Martelli CM, Ximenes RA, Araújo TV, Rocha MA, Ramos RC, et al. Initial description of the presumed congenital Zika syndrome. Am J Public Health. 2016;106:598–600. 10.2105/AJPH.2016.303115 26959258 PMC4816005

[6] Coyne CB, Lazear HM. Zika virus - reigniting the TORCH. Nat Rev Microbiol. 2016;14:707–15. 10.1038/nrmicro.2016.125 27573577

[7] Castanha PMS, Cordeiro MT, Martelli CM, Souza WV, Marques ET Jr, Braga C. Force of infection of dengue serotypes in a population-based study in the northeast of Brazil. Epidemiol Infect. 2013;141:1080–8. 10.1017/S0950268812001367 22800513 PMC9151878

[8] World Health Organization. Dengue vaccine: WHO position paper – July 2016. Wkly Epidemiol Rec. 2016;91:349–64.27476189

[9] Lanciotti RS, Kosoy OL, Laven JJ, Velez JO, Lambert AJ, Johnson AJ, et al. Genetic and serologic properties of Zika virus associated with an epidemic, Yap State, Micronesia, 2007. Emerg Infect Dis. 2008;14:1232–9. 10.3201/eid1408.080287 18680646 PMC2600394

[10] Allwinn R, Doerr HW, Emmerich P, Schmitz H, Preiser W. Cross-reactivity in flavivirus serology: new implications of an old finding? Med Microbiol Immunol (Berl). 2002;190:199–202. 10.1007/s00430-001-0107-9 12005333

[11] Mansfield KL, Horton DL, Johnson N, Li L, Barrett AD, Smith DJ, et al. Flavivirus-induced antibody cross-reactivity. J Gen Virol. 2011;92:2821–9. 10.1099/vir.0.031641-0 21900425 PMC3352572

[12] Crill WD, Chang G-JJ. Localization and characterization of flavivirus envelope glycoprotein cross-reactive epitopes. J Virol. 2004;78:13975–86. 10.1128/JVI.78.24.13975-13986.2004 15564505 PMC533943

[13] de Alwis R, Beltramello M, Messer WB, Sukupolvi-Petty S, Wahala WM, Kraus A, et al. In-depth analysis of the antibody response of individuals exposed to primary dengue virus infection. [Erratum in: PLoS Negl Trop Dis. 2011;5] [8]. PLoS Negl Trop Dis. 2011;5:e1188. 10.1371/journal.pntd.0001188 21713020 PMC3119640

[14] Kostyuchenko VA, Lim EX, Zhang S, Fibriansah G, Ng TS, Ooi JS, et al. Structure of the thermally stable Zika virus. Nature. 2016;533:425–8. 27093288 10.1038/nature17994

[15] Barba-Spaeth G, Dejnirattisai W, Rouvinski A, Vaney MC, Medits I, Sharma A, et al. Structural basis of potent Zika-dengue virus antibody cross-neutralization. [Erratum in: Nature. 2016;539:314. PMID: 27338953]. Nature. 2016;536:48–53. 10.1038/nature18938 27338953

[16] Priyamvada L, Quicke KM, Hudson WH, Onlamoon N, Sewatanon J, Edupuganti S, et al. Human antibody responses after dengue virus infection are highly cross-reactive to Zika virus. Proc Natl Acad Sci U S A. 2016;113:7852–7. 10.1073/pnas.1607931113 27354515 PMC4948328

[17] Stettler K, Beltramello M, Espinosa DA, Graham V, Cassotta A, Bianchi S, et al. Specificity, cross-reactivity, and function of antibodies elicited by Zika virus infection. Science. 2016;353:823–6. 10.1126/science.aaf8505 27417494

[18] Swanstrom JA, Plante JA, Plante KS, Young EF, McGowan E, Gallichotte EN, et al. Dengue virus envelope dimer epitope monoclonal antibodies isolated from dengue patients are protective against Zika virus. MBio. 2016;7:e01123–16. 10.1128/mBio.01123-16 27435464 PMC4958264

[19] Zhao H, Fernandez E, Dowd KA, Speer SD, Platt DJ, Gorman MJ, et al. Structural basis of Zika virus–specific antibody protection. Cell. 2016;166:1016–27. 10.1016/j.cell.2016.07.020 27475895 PMC4983199

[20] Dick GW, Kitchen SF, Haddow AJ. Zika virus. I. Isolations and serological specificity. Trans R Soc Trop Med Hyg. 1952;46:509–20. 10.1016/0035-9203(52)90042-4 12995440

[21] Haddow AD, Schuh AJ, Yasuda CY, Kasper MR, Heang V, Huy R, et al. Genetic characterization of Zika virus strains: geographic expansion of the Asian lineage. PLoS Negl Trop Dis. 2012;6:e1477. 10.1371/journal.pntd.0001477 22389730 PMC3289602

[22] Baronti C, Piorkowski G, Charrel RN, Boubis L, Leparc-Goffart I, de Lamballerie X. Complete coding sequence of zika virus from a French polynesia outbreak in 2013. Genome Announc. 2014;2:e00500–14. 10.1128/genomeA.00500-14 24903869 PMC4047448

[23] Lanciotti RS, Lambert AJ, Holodniy M, Saavedra S, Signor LC. Phylogeny of Zika Virus in Western Hemisphere, 2015. Emerg Infect Dis. 2016;22:933–5. 10.3201/eid2205.160065 27088323 PMC4861537

[24] Brien J D, Lazear HM, Diamond MS. Propagation, quantification, detection, and storage of West Nile virus. Curr Protoc Microbiol. 2013;31:15D.3.1–15D.3.18. 24510289 10.1002/9780471729259.mc15d03s31

[25] Oliphant T, Nybakken GE, Engle M, Xu Q, Nelson CA, Sukupolvi-Petty S, et al. Antibody recognition and neutralization determinants on domains I and II of West Nile Virus envelope protein. J Virol. 2006;80:12149–59. 10.1128/JVI.01732-06 17035317 PMC1676294

[26] Henchal EA, Gentry MK, McCown JM, Brandt WE. Dengue virus-specific and flavivirus group determinants identified with monoclonal antibodies by indirect immunofluorescence. Am J Trop Med Hyg. 1982;31:830–6. 6285749 10.4269/ajtmh.1982.31.830

[27] de Alwis R, Smith SA, Olivarez NP, Messer WB, Huynh JP, Wahala WM, et al. Identification of human neutralizing antibodies that bind to complex epitopes on dengue virions. Proc Natl Acad Sci U S A. 2012;109:7439–44. 10.1073/pnas.1200566109 22499787 PMC3358852

[28] Gallichotte EN, Widman DG, Yount BL, Wahala WM, Durbin A, Whitehead S, et al. A new quaternary structure epitope on dengue virus serotype 2 is the target of durable type-specific neutralizing antibodies. MBio. 2015;6:e01461–15. 10.1128/mBio.01461-15 26463165 PMC4620467

[29] de Alwis R, de Silva AM. Measuring antibody neutralization of dengue virus (DENV) using a flow cytometry-based technique. Methods Mol Biol. 2014;1138:27–39. 10.1007/978-1-4939-0348-1_3 24696329

[30] Kraus AA, Messer W, Haymore LB, de Silva AM. Comparison of plaque- and flow cytometry-based methods for measuring dengue virus neutralization. J Clin Microbiol. 2007;45:3777–80. 10.1128/JCM.00827-07 17804661 PMC2168473

[31] Putnak R, Barvir DA, Burrous JM, Dubois DR, D’Andrea VM, Hoke CH, et al. Development of a purified, inactivated, dengue-2 virus vaccine prototype in Vero cells: immunogenicity and protection in mice and rhesus monkeys. J Infect Dis. 1996;174:1176–84. 10.1093/infdis/174.6.1176 8940206

[32] Maeda A, Maeda J. Review of diagnostic plaque reduction neutralization tests for flavivirus infection. Vet J. 2013;195:33–40. 10.1016/j.tvjl.2012.08.019 23036176

[33] Wahala WMPB, Silva AM. The human antibody response to dengue virus infection. Viruses. 2011;3:2374–95. 10.3390/v3122374 22355444 PMC3280510

[34] Faria NR, Azevedo RS, Kraemer MU, Souza R, Cunha MS, Hill SC, et al. Zika virus in the Americas: Early epidemiological and genetic findings. Science. 2016;352:345–9. 10.1126/science.aaf5036 27013429 PMC4918795

[35] Dejnirattisai W, Wongwiwat W, Supasa S, Zhang X, Dai X, Rouvinski A, et al. A new class of highly potent, broadly neutralizing antibodies isolated from viremic patients infected with dengue virus. Nat Immunol. 2015;16:170–7. 10.1038/ni.3058 25501631 PMC4445969

[36] Corbett KS, Katzelnick L, Tissera H, Amerasinghe A, de Silva AD, de Silva AM. Preexisting neutralizing antibody responses distinguish clinically inapparent and apparent dengue virus infections in a Sri Lankan pediatric cohort. J Infect Dis. 2015;211:590–9. 10.1093/infdis/jiu481 25336728 PMC4375390

[37] Crump JA, Gove S, Parry CM. Management of adolescents and adults with febrile illness in resource limited areas. BMJ. 2011;343(aug08 2):d4847. 10.1136/bmj.d4847 21824901 PMC3164889

[38] Bogoch II, Brady OJ, Kraemer MU, German M, Creatore MI, Brent S, et al. Potential for Zika virus introduction and transmission in resource-limited countries in Africa and the Asia-Pacific region: a modelling study. Lancet Infect Dis. 2016;16:1237–45. 10.1016/S1473-3099(16)30270-5 27593584 PMC5086423

[39] Food and Drug Administration. FDA warns health care providers against relying solely on Zika virus serological IgM assay results; reminds them to wait for confirmatory test results before making patient management decisions [cited 2017 Jan 20]. https://www.fda.gov/MedicalDevices/Safety/AlertsandNotices/ucm534515.htm

[40] Centers for Disease Control and Prevention. Guidance for US laboratories testing for Zika virus infection [cited 2017 Jan 22 ]. https://www.cdc.gov/zika/laboratories/lab-guidance.html

[41] Rabe IB, Staples JE, Villanueva J, Hummel KB, Johnson JA, Rose L, et al.; MTS. MTS. Interim guidance for Interpretation of Zika virus antibody test results. MMWR Morb Mortal Wkly Rep. 2016;65:543–6. 10.15585/mmwr.mm6521e1 27254248

[42] Landry ML, St George K. Laboratory diagnosis of Zika virus infection. Arch Pathol Lab Med. 2017;141:60–7. 10.5858/arpa.2016-0406-SA 27763787

[43] Wilder-Smith A, Gubler DJ. PUBLIC HEALTH. Dengue vaccines at a crossroad. Science. 2015;350:626–7. 10.1126/science.aab4047 26542552

[44] Paulke-Korinek M, Kollaritsch H, Kundi M, Zwazl I, Seidl-Friedrich C, Jelinek T. Persistence of antibodies six years after booster vaccination with inactivated vaccine against Japanese encephalitis. Vaccine. 2015;33:3600–4. 10.1016/j.vaccine.2015.05.037 26036947

[45] Wittermann C, Izu A, Petri E, Gniel D, Fragapane E. Five year follow-up after primary vaccination against tick-borne encephalitis in children. Vaccine. 2015;33:1824–9. 10.1016/j.vaccine.2015.02.038 25728316

[46] Gotuzzo E, Yactayo S, Córdova E. Efficacy and duration of immunity after yellow fever vaccination: systematic review on the need for a booster every 10 years. Am J Trop Med Hyg. 2013;89:434–44. 10.4269/ajtmh.13-0264 24006295 PMC3771278

