# Supplementary material for: Lack of Durable Cross-Neutralizing Antibodies Against Zika Virus from Dengue Virus Infection
Source: Emerg Infect Dis. 2017 May;23(5):773–81. doi: 10.3201/eid2305.161630 (PMC5403059; doi:10.3201/eid2305.161630)
Supplement: Technical Appendix — Limited cross-neutralization between Zika virus; dengue virus and different Zika virus strains with similar neutralization profiles. [file 16-1630-Techapp-s1.pdf]

Article DOI: http://dx.doi.org/10.3201/eid2304.161630

Lack of Durable Cross-Neutralizing Antibodies Against Zika Virus from Dengue Virus Infection

Technical Appendix

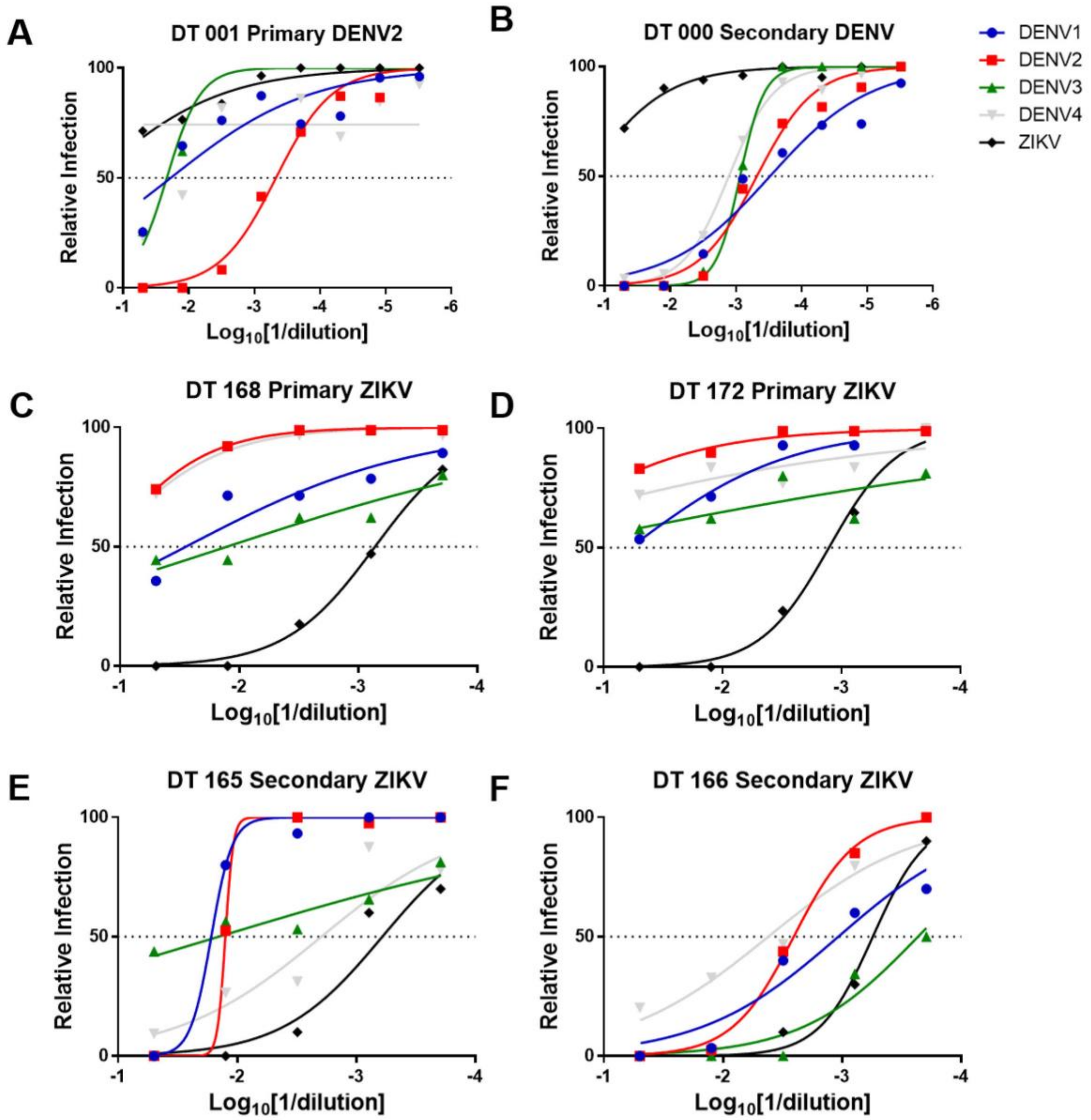

Technical Appendix Figure 1. Limited cross-neutralization between Zika virus and DENV. Neutralization activity of immune serum from primary DENV (A), secondary DENV (B), primary Zika virus (C and D), or secondary Zika virus (E, F) against Zika virus H/PF/2013 and 4 serotypes of DENV was determined by

focus-reduction neutralization test (A, B) or plaque reduction neutralization test (C–F) on Vero cells. Neutralizing activity is displayed as a percentage of the number of foci or plaques counted for naïve human serum at each dilution. Results represent the mean of technical replicates per sample and are representative of at least 2 repeats for each serum sample. DENV, dengue virus.

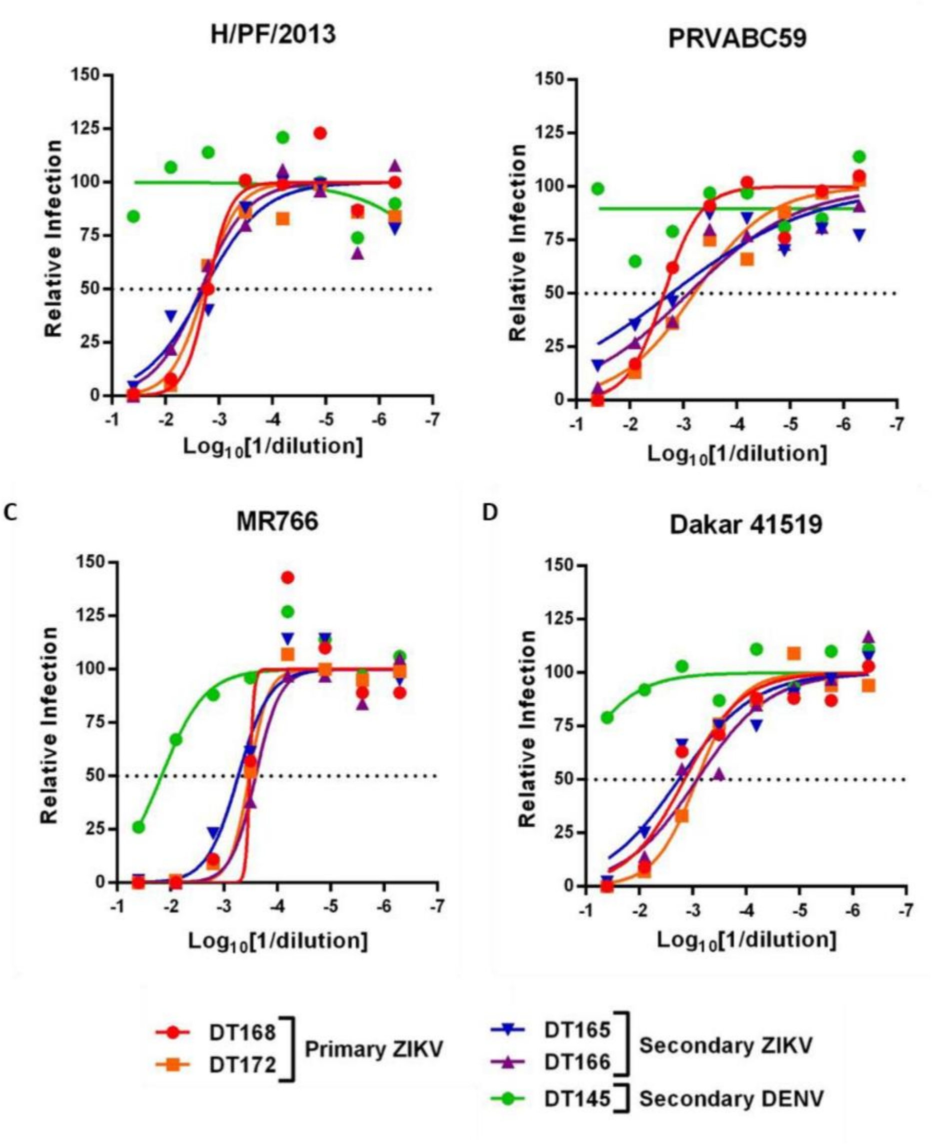

Technical Appendix Figure 2. Different Zika virus strains have similar neutralization profiles. Neutralization of 4 Zika virus strains, H/PF/2013 (A), PRVABC59 (B), MR766 (C), and Dakar 41519 (D) by human immune serum was determined by focus-reduction neutralization test on Vero cells.
